# A Review of the Implications of Heterozygosity and Inbreeding on Germplasm Biodiversity and Its Conservation in the Silkworm, *Bombyx mori*

**DOI:** 10.1673/031.011.0108

**Published:** 2011-01-26

**Authors:** A.H. Jingade, K. Vijayan, P. Somasundaram, G.K. Srivasababu, C.K. Kamble

**Affiliations:** ^1^ Central Sericultural Germplasm Resources Centre, Central Silk Board, Thally Road, Hosur, Tamil Nadu-635 109, India; ^2^ Institute of Plant and Microbial Biology, Academia Sinica, Taipei, Taiwan-115, ROC.

**Keywords:** conservation, genetic diversity, inbreeding depression, molecular markers

## Abstract

Silkworm genebanks assume paramount importance as the reservoirs of biodiversity and source of alleles that can be easily retrieved for genetic enhancement of popular breeds. More than 4000 *Bombyx mori* L (Lepidoptera: Bombycidae) strains are currently available and these strains are maintained through continuous sibling mating. This repeated sibling mating makes the populations of each strain more homozygous, but leads to loss of unique and valuable genes through the process of inbreeding depression. Hence, it is essential to maintain a minimal degree of heterozygosity within the population of each silkworm strain, especially in the traditional geographic strains, to avoid such loss. As a result, accurate estimation of genetic diversity is becoming more important in silkworm genetic resources conservation. Application of molecular markers help estimate genetic diversity much more accurately than that of morphological traits. Since a minimal amount of heterozygosity in each silkworm strain is essential for better conservation by avoiding inbreeding depression, this article overviews both theoretical and practical importance of heterozygosity together with impacts of inbreeding depression and the merits and demerits of neutral molecular markers for measurements of both heterozygosity and inbreeding depression in the silkworm *Bombyx mori.*

## Introduction

The mulberry silkworm, *Bombyx mori* L. (Lepidoptera: Bombycidae) is a completely domesticated lepidopteran insect that has been used for silk production for more than 5000 years (International Silkworm Genome Consortium 2008). Its production base is spread over 60 countries with Asian countries producing over 90% of mulberry silk production (Datta and Nanavathy 2007). In addition to being used in silk industry, *B. mori* is also used for production of recombinant proteins (Tamura et al. 2000; Tomita et al. 2003) and for basic research in microbiology, physiology, and genetics (Wills et al. 1995). Considering the great economic importance attached to *B. mori,* the traditional and leading silk producing countries such as China, Japan, India, Russia, Korea, and Bulgaria have collected a number of silkworm races suitable for a wide range of agro-climatic conditions (Tables 1, 2). Currently more than 4000 strains are available in the germplasm of *B. mori* (Nagaraju 2002; Kumaresan et al. 2004a, 2004b), which includes uni-, bi-, and polyvoltine varieties. These silkworm strains have been maintained under *ex situ* conditions through years of continuous sibling mating and selection. Consequently, these strains have possibly undergone many evolutionary changes through mutations (natural as well as man-made selections), thereby creating a wide genetic diversity among the strains; but this diversity becomes more homogenous within the strain. This repeated inbreeding in each traditional strain results in the loss of valuable genes through a process called inbreeding depression. Therefore, it is imperative to prevent this genetic erosion due to excessive inbreeding to conserve the genetic resources of geographical strains for their use in divergent breeding programmes. In this context it is worth bearing in mind that the process of conservation of the genetic resources of these strains does not only necessitate acquisition and physical possession of them, it is equally important to ensure that the original genetic characters are maintained and the rare and endemic strains are well protected from extinction. Therefore, all possible measures need to be taken to preserve the originality of the *B. mori* races as the gene banks are reservoirs of gene assemblies consisting of geographically isolated races, genetic stocks, and breeds collected from indigenous and exotic regions. Frankel (1984) developed the concept of core collections to preserve germplasm accessions with minimum repetitiveness and at the same time retain most of its original genetic diversity. This concept was further elaborated by Frankel and Brown (1984). Rapid replacement of traditional races by high yielding breeds/hybrids also threatens the very survival of a number of old races, which are the result of conscious or subconscious selection over a long period of time. Some of the old Indian traditional silkworm races such as Chotopolu and ancient breeds viz., Nistid, Nismo, Ichat, and Itan are either extinct or on the verge of extinction due to the absence of a proper conservation system (Thangavelu 1998). It is, therefore, high time for silkworm conservationists and geneticists to take appropriate measures to protect their valuable resources from further degradation and extinction.

**Table 1.**
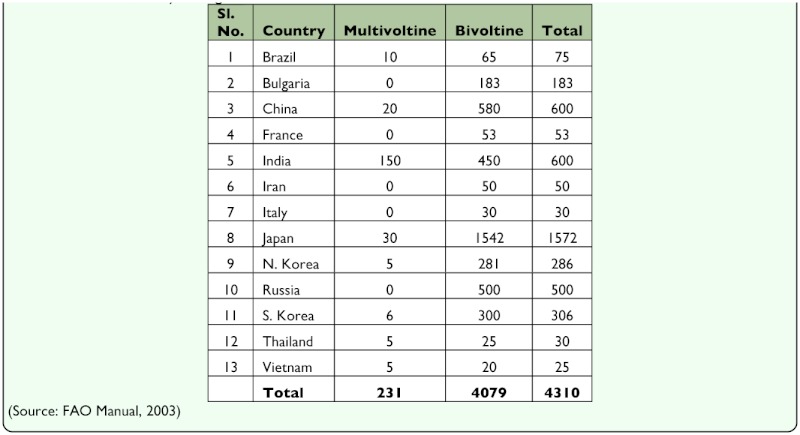
Silkworm, *Bombyx mori* genetic resources available in different countries

**Table 2.**
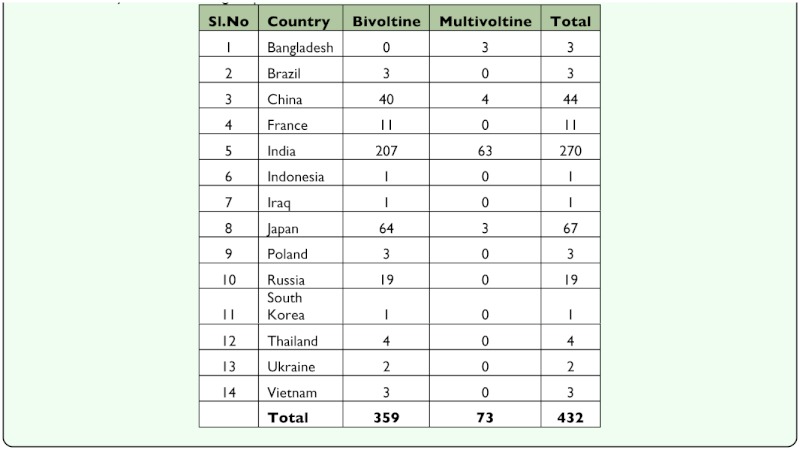
Country-wide silkworm germplasm maintained at Central Sericulture Resources Centre, Hosur, Tamil Nadu, India

### Genetic diversity in the silkworm *Bombyx mori*

Genetic diversity is the genetic variation within species, both among geographically separated populations and among individuals within a single population. Genetic diversity is an essential aspect in conservation biology because a fundamental concept of natural selection states that the rate of evolutionary change in a population is proportional to the amount of genetic diversity present in it (Fisher 1930). Decreasing genetic diversity increases the extinction risk of populations due to a decline in fitness. Therefore, both biochemical and molecular markers have recently been employed to estimate the extent of genetic diversity present among various types of silkworm strains such as mono-, bi-and multivoltines present in China, Japan, Korea, India, and several other countries. For instance, Nagaraja and Nagaraju (1995) estimated genetic diversity among thirteen silkworm strains from tropical and temperate regions. The study placed the silkworms into two distinct groups consisting of six diapausing and seven non-diapausing genotypes. The genotype, Pure Mysore, was found to be the most divergent genotype among the genotypes studied. Likewise, Chatterjee and Pradeep (2003), Dalirsefat et al (2009), Li et al. (2005), Lu et al. (2002), Nagaraju et al. (2001), Prasad et al. (2005), Reddy et al. (1999a, b), and Xia et al. (1998) used AFLP, RFLP, ISSR, and SSR markers to estimate the genetic distance among silkworm strains that differed in several morphological traits. Pairwise genetic diversity estimation among 20 mutant strains by Velu et al. (2008) revealed 0.045 to 0.553 genetic diversity and consequent grouping among the silkworms. Recently Xia et al (2009) constructed a single-base pair resolution genetic map using genomic information from 40 domesticated and wild silkworm strains. With the help of 16 million single nucleotide polymorphism (SNP) markers, identified from the total genomic information, the domestication events and subsequent genetic differentiation in *B. mori* have been worked out. The study revealed significant genetic divergence between wild and domesticated strains of *B. mori.* The genetic variation was measured using the population size scaled mutation rate which was significantly smaller in domesticated strains (0.011) when compared to the wild strains (0.013). The rate of heterozygosity in domesticated strains was two times lower than that in wild varieties (0.003 and 0.008, respectively), which is most likely attributed to inbreeding experienced by domesticated lines. The study also shows clear genetic separation between domesticated and wild varieties and relatively less gene flow between them.

### Significance of heterozygosity and inbreeding depression in silkworm race maintanance

Heterozygosity is the state of having different alleles with regard to a given character. Allelic diversity, on the other hand, determines a population's ability to respond to long-term selection over many generations, and ultimately the survival of the population (perhaps even of the species). Individual heterozygosity is expected to be correlated between parents and their offspring. The expected correlation is maximal, approaching r = 0.50, when allelic frequencies are highly asymmetric, and it is zero when the allelic frequencies are equal to 0.50 (Mitton et al. 1993). Inbreeding is the process of mating of two closely related individuals. The inbreeding coefficient of an individual refers to how closely related its parents are. The major effect of inbreeding is the reduction in mean performance of the population. Inbreeding tends to reduce the level of the characters connected with fitness and consequently can lead to loss of general vigour and fertility. This reduced fitness of populations caused by the manifestation of deleterious recessive genes is termed inbreeding depression. ID usually reduces reproductive performance and survival (Falconer 1989), and has been documented in many populations ever since Darwin (1876) described it (Wright 1977; Charlesworth and Charlesworth 1987; Ralls et al. 1988; Falconer 1989).

In *B. mori,* it is speculated that the wild gene pool has undergone considerable selection in every generation during the course of its domestication, thougth it might be unintentional (http://unp.un.org/). Consequently the pool reached its current level of diversity of characteristics within the species. Genetic priciples and breeding techniques of modern genetic science were utilised for cross breeding and exploitation of the phenomenon of hybrid vigour in the silkworm. A high level of heterosis/hybrid vigour for various quantitative characters was found to occur in the crosses of highly inbred lines and geographically divergent populations of the silkworm (Harada 1957; Harada 1961; Krishnaswami et al. 1964; Hirobe 1968; Yokoyama 1979; Gamo and Hirabayashi 1983; Nagaraju 1990). Low heritability traits, such as survival rate, displays extensive heterosis and have become the focal point for exploitation of heterosis in the silkworm (Nagaraju and Pavan Kumar 1995). In silkworms, when an inital choice of parents has to be made to obtain heterosis, it is important to asertain the level of parental divergence (Jolly et al. 1989; Chatterjee et al. 1993; Mukerjee et al. 1999; Kumaresan et al. 2003 a, 2003b). Parental homozygosity has a distinct influence on the degree of manifestation of heterosis (Nagaraju and Goldsmith 2002). Hybrid vigour arises when the crossing of two inbred lines (or an outbred line with an inbred line) produces individuals that are heterozygous at loci that were previously homozygous for deleterious alleles (Falconer 1981). Petkov et al. (1999) has attempted to solve the problem of applying inbreeding to the silkworm. However the evidence of effects of inbreeding on inbreeding coefficients in silkworm populations is limited.

### Estimation of inbreeding depression

Based on the Hardy and Weinberg law, which states that in large random mating populations, all the genotypes have equal fitness, i.e. there is no selection, therefore both allelic and genotypic frequencies in the population remain constant as long as other disturbing factors such as non-random mating, mutations, selection, limited population size, random genetic drift, and gene flow do not act (Hardy 1908). Wright (1922) formulated F-statistics to measure the inbreeding coefficient ‘F’ at a locus using the formula *2pq (l - F)* where *p* and *q* are the allelic frequencies at a diallelic locus and F is the inbreeding coefficient. Individuals with the same expected inbreeding co-efficients vary in their degree of homozygosity hence there is opportunity for natural selection to modify the array of genotypic frequencies, particularly to favour the more heterozygous individuals. Inbreeding depression for a trait is a linear function of the inbreeding coefficient (Lynch and Walsh 1998). Inbreeding depression has been measured at the population level by comparing the mean phenotypes of inbred and outbred individuals. Inbreeding depression is estimated from the difference in mean fitness between inbred and outbred individuals within the same family, often the progeny of a single maternal parent. Different techniques are available to determine the level of inbreeding. The advantages and disadvantages of different approaches for measuring inbreeding and inbreeding depression is given in Table 3. Genetic drift (F_ST_), quantified by effective population size, is another parameter with which inbreeding depression within a population is measured. *F_ST_* measures the decrease in heterozygosity due to the finite size of the population and the effective size. In fact *F_ST_* gives a measure of the genetic drift among conceptual replicates of the population in an infinite nonrandom mating population, *F_ST_*= 0 and *F_IT_*= *F_IS_,* rising toward a value which depends on the proportion of inbred matings. Thus, genetic drift *(F_ST_)* and decline in heterozygosity *(F_IT_)* will have the same rate of change except that the drift runs one generation ahead of the decline in heterozygosity.

In countries that practice sericulture, several pure races belonging to different voltinism groups are maintained in germplasm centres by selection and inbreeding. Sibling mating of the progenies derived from a single brood is preferred for pure stocks so that the original traits of the races are maintained through generations. The homozygous races maintained in the germplasm are an important source of breeding new silkworm breeds through hybridization (Subramanya and Bishop 2009). Generally breeders try to maintain the original characters of the races through selection with care to avoid inbreeding depression. Although, much work has been carried out on quantitative traits such as yield and biochemical parameters (Chatterjee et al. 1993; Natcheva et al. 2001; Nagaraju 2002) not much work has been done on the impacts of inbreeding on quantitative traits. Two statistical models, viz. general mixed model and a fixed model, are available for estimating the impact of inbreeding on quantitative traits (Subramanya and Bishop 2009).

**Table 3.**
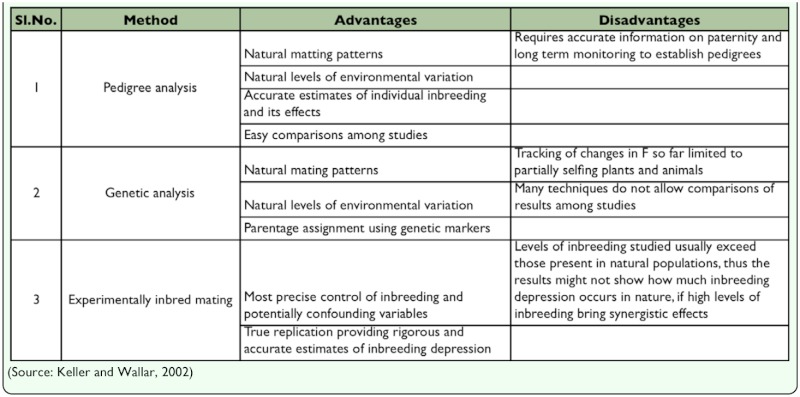
Advantages and disadvantages of approaches for measuring inbreeding and inbreeding depression

### Inbreeding depression in silkworm races

The general procedure of silkworm breeding is the judicious selection of parents and their subsequent matings to meet definite purposes. Genetic improvement of silkworm races is generally achieved by conventional breeding methods of hybridization between selected parents combined with selection for desired traits. The improvement of silkworm strains has been achieved over a long period by pure line selections. The advantage of using F1 hybrids for commercial exploitation was emphasized by Toyama as early as in 1906. Inbred lines are developed by crossing between two strains showing excellent characteristics and subsequent sibling-mating and progeny selections for seven or more generations. Silkworm strains maintained at germplasms serve as genetic stocks. Genetic stock refers to an inbred stock that carries one or several qualitative characters. A parental pure line refers to an inbred stock that can be crossed with other purelines in order to produce a hybrid variety. This is done in order to exploit heterosis or the hybrid vigour phenomena of quantitative characters and qualitative traits that meet the needs and interests of breeders. Parents that are complementary for desired characters in one line are selected and a cross is made. Sibling-mating is carried out within an egg batch of the F1, and the individual F2 silkworm pairs are selected with each maintained as a separate family through F5 or F6. General combining ability with a topcross tester is tested when uniformity is found to be necessary. The best general combiners are selected to identify strains used recurrently in a series of breeding programmes and specific crosses are made and tested for combining ability.

The most intense form of inbreeding in the silkworm is full-siblingmating. Less intense forms of inbreeding occurs between cousins, second cousins, and other types of family matings. The relative effects can be seen in the inbreeding coefficient, F, in consecutive generations. For the mating type of full-sib, inbreeding coefficients increase from 0.25, 0.37, 0.50, 0.59, and up to 0.67 as inbred generations proceed from generation 1, 2, 3, 4, and go up to 5, respectively. In practical terms, the breeding expectation is that inbreeding leads to isolation of homozygous lines with reduced vigor. The reduction in vigor is not in a direct relationship with generation, but generally parallels the reduction in heterozygosity. Inbreeding can be expedited by selecting for the ultimate inbred characters, if they are defined. That is, if the inbred characteristic is round cocoon and long larval duration, selection of such individuals will give a better chance for achieving a relatively high degree of homozygosity. If characters are undefined, the breeder should select against heterotic properties (http://unp.un.org/). The success and spread of silkworm rearing was mostly due to the use of F1 silkworm hybrids of exotic temperate silkworm male parental strains and native female tropical silkworm strains (Nagaraju 2002). Continuous inbreeding results in the accumulation of many deleterious genes leading to inbreeding depression, thereby resulting in the deterioration of some commercial characters. However, the overall combination of beneficial traits could be achieved in a reasonable way by employing inbreeding techniques coupled with selection as the silkworm breeder has at his disposal a diversified array of gene combination to manipulate and isolate new silkworm breeds having desirable qualities for commercial exploitation (Raju and Krishnamurthy 1993). The improvement of indigenous breed could be achieved through hybridizations utilizing exotic breeds (Kovalov 1970). Inbreeding the hybrids to isolate and stabilize silkworm breeds for the desired traits has been well documented by many breeders (Hirobe 1968; Kovalov 1970; Gamo 1976). Artificial selection has been widely utilised in the breeding programmes concerning this commercially important insect. Selection increases the frequency of homozygotes and makes homozygous effects stronger (Whitlock 2002). In *B. mori,* qualitative changes in the genetic basis, induced by artificial selection, resulted in genetically divergent populations with different phenotypic values of larval duration. In the Nistari strain of *B. mori,* where short larval duration is part of fitness component, when artificial selection was imposed the selection pressure acted against this fitness component and caused longer larval duration inbreed lines by favouring variation at the multilocus level thus causing genetic divergence (Pradeep et al. 2005).

### Molecular markers for heterozygosity estimation

Heterozygosity fitness correlations (HFC) historically appeared as an opportunity to develop marker-based genetics, an approach boosted by the discovery of allozyme polymorphism (Lewontin and Hubby 1966). Geneticists had known since as early as 1910–1920 that manipulations of genomic heterozygosity (using inbred lines) could affect fitness components and crop yields (Shull 1952). HFC provided a potential ‘microscopic’ basis of the ‘macro-scopic’ effects observed using inbred strains. Although the relationship between heterozygosity at the genome level and fitness was not in doubt, the question came in identifying the particular genes involved and how they affected fitness. Genes showing HFC were obviously good candidates. HFC is quantified by the variance in a fitness trait explained by its regression on heterozygosity *(r *^2^). Most estimates yield an order of magnitude of 0.01–0.05. However, sample size *(N)* must be considered as, even if the true correlation is zero, an average value of *1/(N-*1*)* is expected by chance (Sokal & Rohlf 1995). As most samples are of the order 10^2^, *1/(N-*1*)* is of the same magnitude as the r^2^ values observed. For multiple regressions (*L* loci as separate ‘heterozygosity’ variables) the null expectation for *R^2^* is *L*/*(N-1),* a serious problem if *L* is not <*N* (Bush et al. 1987). However, the overall lack of significant correlations in natural populations of such a model organism as *Drosophila* (Houle 1989) and in many other species, even among bivalves or pines (Britten 1996), strongly suggests that HFC is not universal. For a long time allozymes have been the most reliable and convenient genetic markers. This situation is now changing with the development of PCR-based codominant markers such as microsatellites, anonymous RFLP and intron length polymorphisms (Mitton 1994). Heterozygosity may be underestimated due to lack of resolution (alleles with similar mobility appear as the same allele), somatic aneuploidy, or null alleles. Most PCR-based markers also have null alleles. Most molecular markers have the same inherent defects as allozymes. However, PCR markers, unlike allozymes, allow scoring of very small organisms (such as bivalve larvae) at many poly-morphic loci. Polymorphic markers (microsatellites) seem unsuitable as almost all individuals will be heterozygous. However, microsatellites provide estimates of the quantity of divergence between two alleles (specifically, the difference in repeat number, under a stepwise mutation model), an interesting alternative to the traditional heterozygosity (in which alleles are classified as either identical or different). Moreover molecular markers can be used in conjunction with allozymes, in order to compare the latter with *a priori* neutral loci. Such experiments allow evaluation of possible causes of HFC other than null alleles or aneuploidy.

Assessment of heterozygosity in each silkworm race is important for efficient management and conservation of genetic resources (Graner et al. 2004). Genotyping-based approaches (Table 4) can be used to quantify levels of inbreeding and to gain a better understanding of the genetic architecture of inbreeding depression through the detection of genes that affect it. Although not much effort has been made to measure the heterozygosis in different strains of *B. mori,* the few attempts which made recently have revealed that within strains genetic diversity is much less than it is among the strains. Staykova (2008) used isozyme patterns of 480 individuals of eight silkworm strains to assess the heterozygosity among the silkworm populations. The heterozygosity among the strains varied from 0.099 in China to 0.238 in Kinshu. Further, the study also revealed that the observed heterozygosity was much less than the expected value, which was atributed to the effect of the inbreeding. Similarly, using molecular markers such as AFLP in 23 silkworm strains composed of 12 Japanese and 11 Chinese strains, Gavria et al (2006) showed that Japanese stains are more heterogenous than the Chinese strains. However, a recent study with three Iranian and three Japanese silkworm strains revealed low within-strain genetic variation (26.6%) and high genetic diversity between strains (75.4%). Among the strains, within strain variation was least in the Japanse strain P107 (0.08), and highest in the Iranian Khorasan Orange strain (0.18) (Dalirsefat et al. 2007). Since these studies are with isozyme (less polymorphism) and dominant molecular markers (AFLP), better depth of the heterozygosity cannot be revealed. Codominant markers that can reveal both homozygous and heterozygous loci have to be used. The most commonly used PCR based co-dominant markers are microsatellites (SSR) and single nucleotide polymorphisms (SNPs). Since a number of SSR primers have been developed from silkworm genome (Reddy et al. 1999a) they can be used for assessing the loss of heterozygosity in subsequent generations. SNPs is a new generation marker system, and represents stable, diallelic nucleotide variations that are distributed throughout the genome and are the most abundant resource for analysis of genetic variation (Kwok et al. 1994). Generally, microsatellites provide a higher level of resolution because of the large number of alleles per locus. But SNPs provide large data sets because of high abundance in the genome and high throughput genotyping methods. To detect inbreeding depression, the level of heterozygosity is correlated with one or more measures of fitness. Modern genomic technologies enable screening of a large genome-wide set of molecular markers, which give a reliable estimate of F1. In combination with fitness measures, the genomic data also enable detection of genes associated with inbreeding depression (Kristensen et al. 2010). The results of SNPs can provide a fundamental description of the nucleotide diversity of the silkworm genome (Cheng et al. 2004), which is evident from the results reported by Xia et al (2009). Using information from 16 million SNPs identified in genomes of 40 silkworm strains, they estimated the heterozygosity in wild and domesticated silkworm strains. The rate of heterozygosity in the wild silkworm (0.0080) was more than double of that present in domesticated strains (0.0032). This significant reduction in the heterozygosity of the domesticated strains was attributed to inbreeding or the population-size bottle neck experience by the domesticated strains, as a 90% reduction in the effective population size occurred due to domestication.

**Table 4.**
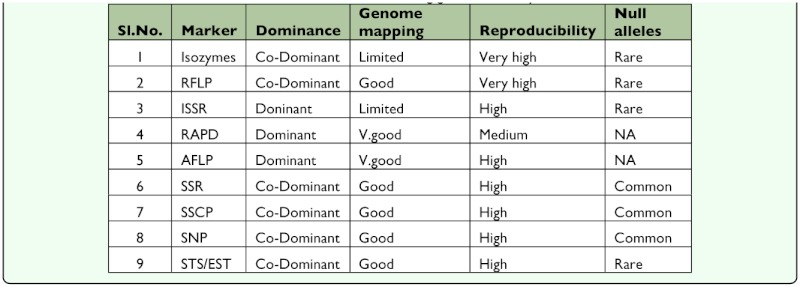
Characteristics of different molecular markers for assessing genetic diversity

### Restoration of genetic diversity in silkworm races

*B. mori* is a completely domesticated insect and has undergone intense artificial selection, (Goldsmith 2005). Therefore, it has to depend on humans for its survival. Years of continuous domestication have made *B. mori* unable to survive in the wild. Therefore, natural selection induced genetic diversity is limited to few traits such as voltinism. Hence, it is essential to conserve and utilize the wild relatives of *B. mori* to broaden the genetic base of the domesticated races apart from utilizing the geographically divergent races, mutants, sex-limited races, evolved breeds, breeder's genetic stocks, and exotic resources. The wild relatives of *Bombyx* are ecologically very vulnerable and the vulnerability at different spatial and temporal scales is not known. The design of biodiversity network in sericulture involving the complementaries of wild relatives and domesticated *B. mori* is also not well established. Therefore, conservation of wild as well as domesticated seribiodiversity resources is very essential for sustainable development of sericulture since loss of genetic resources of domesticated and wild relatives of *Bombyx* species along with their unique genes may be disadvantage for future generations (FAO Manual 2003).

## Summary

With the advent of co-dominant DNA markers that are superior to allozymes, the identification of heterozygosity among the populations of races, variety, or breeds of a species became easier. Use of SSR and SNP markers to identify the heterozygosity levels for conservation of genetic material for true- to-type genotype maintenance among germplasm stocks has become a regular practice. The use of DNA markers to relate genetic homozygosity and genetic distance of the parental strains to the manifestation of hybrid vigour, and understanding the genetic basis of heterosis, will be quite useful to select suitable parental lines for hybridization programmes. Considering the large number of silkworm inbred lines that are maintained in the silkworm breeding stations, it is prudent to use molecular markers to identify parental lines that are most suitable for developing heterotic hybrids. Silkworm strain-specific markers could also be used for authentication of races, protection of breeders' rights, and periodical monitoring of the purity of varieties in the conservation centres. So far, evidence of effects of inbreeding on inbreeding coefficients in silkworm populations is limited. Hence understanding the effects of inbreeding for various traits can be very crucial points in the management of germplasm. Another important objective of silkworm genetic resource conservation is to protect the available seri-biodiversity from extinction. At present only few old Indian indigenous races survive, viz. Barapolu, Chotapolu, Nistari, Sarupat, and Moria; several indigenous races like Ichot, Nishmo, Itan, and the univoltine Kashmiri races are extinct (FAO Manual 2003). Therefore, it is essentaial to develop appropriate strategies for the conservation of biodiversity not only for the vulnerable populations, but also for sustainable utilisation of silkworm genetic resources. Effective conservation plans need to be developed.

## Abbreviations

HFC,: heterozygosity fitness correlations
ID,: inbreeding depression
SNP,: single nucleotide polymorphisms
SSR,: microsatellites
